# Genetic counseling around the globe: Promoting international cooperation and collaboration

**DOI:** 10.1016/j.gimo.2024.101900

**Published:** 2024-11-22

**Authors:** Kelly E. Ormond, Juliana Mei-Har Lee, Clara L. Gaff

**Affiliations:** 1 ETH-Zurich, Zurich, Switzerland; 2 Stanford University School of Medicine, Stanford, CA; 3 Genetic Counselling Asia, Malaysia; 4 National University of Malaysia, Malaysia; 5 The University of Melbourne, Parkville, Australia

As we detailed in our call for articles^1^ for the special issue “Genetic counseling around the globe,” significant expansion has occurred in the past decade in the profession of genetic counselors and in the provision of genetic counseling services around the globe. Yet, this information has been fragmented across the literature, in various types of journals with varied degrees of accessibility, and often in country- or regional-specific publications. Our hope is that this special issue allows readers to begin to integrate knowledge about genetic counseling across the globe, through articles that profile the progress in genetic counseling around the world over the past decade. Much has changed since prior special issues on the topic, the most recent in 2013 (*Journal of Genetic Counseling* v22:6^2^).

Because our positionality is critical to understanding our interest in the topic of global genetic counseling and our approach to the special issue, we would like to start with a few words about our own background. Between the 3 of us, we have lived and worked on 4 continents (Asia, Australasia, Europe, and North America) and in at least 6 countries and regions (Australia, Hong Kong, Malaysia, Switzerland, United Kingdom, and United States). Collectively, we have ∼80 years of experience in the profession of genetic counseling. At various points in time, each of us has worked in a clinical setting, primarily in teaching hospitals, but J.M.H.L. has also worked in a private practice across several South East Asian countries. We have all been involved in graduate-level training of genetic counselors (in the United States, United Kingdom, Australia, Malaysia, Italy, and Austria) and served as chairs/presidents of professional societies—for example, Human Genetics Society of Australasia’s special interest group the Australasian Society of Genetic Counsellors, the US-based National Society of Genetic Counselors, the Professional Society of Genetic Counselors in Asia, and the Transnational Alliance for Genetic Counselors. Although 2 of us have only practiced clinically in English (C.L.G. and K.E.O.), we have trained individuals whose first languages are not English, and J.M.-H.L. has seen clients in English, Malay, Cantonese, and Mandarin. Each of us has been interested in promoting international cooperation and collaboration around genetic counseling practice and the profession through our various leadership roles.

We received submissions for consideration from around the world (representing Africa, the Americas, Asia, Australasia, and Europe), and we present to you 20 articles that cover a wide range of topics, countries, and stages of development of the genetic counselor profession and genetic counseling as a service. We encountered many challenges putting this special issue together. Expected or unexpected, all are worth considering in the hopes that this special issue provides a foundation for future efforts to progress genetic counseling internationally.

First, a major limitation of this special issue is that all articles needed to be written in English. This may have placed constraints on potential authors; therefore, work from countries where English is a stronger focus may be overrepresented. Hopefully in time, the growing use of artificial intelligence tools such as Grammarly or ChatGPT to assist with English language translations and grammatical checks will help reduce barriers to publication for those for whom English is not their first language. Certainly, the article by Abad et al^3^ demonstrates that the literature on genetic counseling is more difficult to review and synthesize in languages other than English. Second, although open access journals (such as this one) increase the availability of articles to audiences around the world, they often require significant publication-related payments. This can also limit submissions. Nonetheless, we felt that *Genetics in Medicine Open* is a good journal to publish a special issue on this topic, enabling professionals all around the world to access the articles; we worked hard with the journal’s administrators to consider fee waivers for all submissions from countries that were considered newer to genetic counseling or if fees might pose publication challenges. Notwithstanding our efforts, these 2 limitations combine to significantly affect what sorts of articles about the genetic counseling profession and genetic counseling services are ultimately published and read worldwide. We are very aware that this influences how the profession develops over time.

Finally, there is a huge challenge in how academic scholarship develops within a field that is still in early stages of development in many countries. This played out in several ways as the special issue progressed. One major challenge was obtaining peer reviewers for each article—we felt strongly that each article should have at least 1 reviewer from the general region from where it originated (eg, an article with authors from the Middle East should have at least 1 reviewer from that region). This process became challenging for regions where there are fewer professionals with genetic counseling expertise; frequently professionals within those regions had a conflict of interest, such as recent collaborators or prior trainees, having, for example trained the article author or working in the same institution. An additional challenge was identifying reviewers in those regions with research and reviewing experience because there are fewer genetics professionals with specific research training or doctoral degrees. Academic writing and research skills have become more of a focus for genetic counselors and other genetics providers where the profession has a long history of growth and is well established. In other countries, research and academic activities may be a lower priority for genetic counselors working primarily in a clinical setting and in settings with limited numbers of genetic counselors.

The first articles in our special issue by Weil et al^4^ (2024) speaks strongly to the reasons we as editors initiated this special issue. The article asks readers to decenter their views of genetic counseling practice and education from the primarily White-European, American/Western perspectives from which it originated and to acknowledge the diversity in those providing and receiving genetic counseling around the globe. This article sets the stage for the special issue, the remainder of which provides examples of exactly the diversity that Weil et al^4^ describe. Of note, perhaps because of the way we framed the call for articles in this special issue, we did not receive many articles that directly reference cultural influences, although it comes up indirectly in many articles.

Next, we present 10 articles that focus on the status of the genetic counselor profession around the world, showcasing the similarities and differences that exist across service delivery, training and roles of genetic counselors. First, Ormond et al^5^ (2024) summarize the significant growth of the profession from ∼7000 genetic counselors in 2017 to over 10,000 in 2023. This article gives a broad overview of the common struggles and challenges that genetic counselors around the globe face, including topics such as professional recognition and regulation. The next 2 articles describe the situation of genetic counselors in 2 countries where the profession of genetic counselors arose approximately 30 years ago: South Africa (Wessels et al,^6^ 2024) and Australia/New Zealand (Kanga-Parabia, et al,^7^ 2024). These 2 articles juxtapose the quite different pace and outcomes of professional development in the field. For example, in South Africa, which has a population of nearly 60 million, there are ∼30 genetic counselors, whereas in Australasia, which has a population of less than 30 million, there are more than 400. Kanga-Parabia et al^7^ present a workforce status survey from Australasia. This professional status survey demonstrates the maturity of the profession in Australasia, allowing it to focus on diversity in the profession and to recognize the importance of mirroring population diversity in the workforce.

Seven articles consider the status of the genetic counselor profession in countries or regions where the genetic counseling profession is newer or could be considered as emerging. Abad et al^3^ (2024) summarize the literature on genetic counseling published by authors in Asia over the past 50 years. This article gives us insights into how the field and profession, as well as the published literature, are developing over time, highlighting which topics are most relevant to the region. Many of the articles describe countries where the genetic counseling profession is just starting (German-speaking D-A-CH countries,^8^^,^^9^ Qatar,^10^ and Oman^11^), and 2 describe countries where it is identified as a critical need, but the profession has not yet started in a practical manner (Uganda^12^ and Chile^13^). These articles in combination highlight how the establishment of genetic counseling services and the profession of genetic counselors will vary significantly based on local factors, such as the health care system and reimbursement models, governmental policies, and the role of other professionals. They also highlight how early stages of the profession may first require training of professionals outside the country, in areas that have more well-established genetic counseling practice, and then providing local (in-country or in-region) training that can address the medical, social, cultural, religious, and linguistic issues that are critical to successful practice. Both the Austrian program (serving D-A-CH) and the Omani programs also describe educational approaches that partner local clinical training internships with a single training course as a way to optimize training until a country reaches a point where both are sustainable. Finally, many of these articles highlight the still-challenging question of how one defines “who is a genetic counselor” and what that definition is based on.

We then hear from several organizations, each discussing a range of mechanisms of global outreach to further develop the profession of genetic counselors around the globe. Two US-based organizations (SPLAGen,^14^ focusing on Latin America, and an approach with the Pakistan Medical Genetics organization^15^), each founded by persons with ties to the region, are trying to increase access to genetic services and genetic counseling in underserved regions. These different approaches have strengths and limitations but may shape readers’ views on new ways to promote global expansion of genetic counseling.

Training and professional issues were another critical topic, addressed in the next 4 articles. Given that a notable percentage of emerging genetic counseling services are “seeded” by training individuals at more well-established locations (eg, the United States, United Kingdom, Australia, or South Africa), issues such as the language of training^16^ and comparisons of how genetic services are delivered across different countries arise.^17^ We received several interesting articles by trainees or recent graduates detailing their experiences in these areas. These articles should be of interest for genetic counseling educators everywhere. Once in practice, learning does not end—continuing education is critical to stay on top of this fast-moving technology, whereas mentorship helps new professionals have support and supervision to grow into their roles. Valverde et al^18^ present an example training approach for global online continuing education, and Canton et al^19^ present a model of mentorship in Australia; both could be considered by others across the globe as examples of critical action that can be taken to sustain practicing professionals in areas with smaller numbers of experienced genetic counselors.

Finally, although many of the submissions focused on genetic counselors and the profession of genetic counseling, we also present 3 research-focused articles that document creative or novel approaches to delivering genetic counseling services in various locations around the globe: Germany^20^ (cancer genetics services via iKNOW), Mexico^21^ (cancer genetics research studies), and Australia^22^ (rapid genome sequencing in a critical care setting). These approaches are ones that may have wider relevance to genetic counseling service provision around the globe.

As we think about what these articles tell us in combination about the field and profession of genetic counseling, we have several thoughts. First, the majority of the articles we considered for the special issue were (at least initially) deeply grounded in telling the story of the individual country or research study that was being presented. As such, and read in isolation, they provided interesting insights into how practice is occurring and the profession developing locally. However, it is our hope as guest editors that this issue will inspire our colleagues to move beyond awareness and understanding of what is happening around the globe toward a higher level by processing the commonalities and differences in genetic counseling around the globe. In our editorial roles, we have encouraged authors to also consider how their story may inform and assist others navigating the complexities of providing genetic counseling around the globe and many have reframed their articles to include these insights. Through reading about how genetic counseling practice is described internationally, it is evident that the value of client-centered practice is held in common, even while the manner in which it is achieved is the result of region- and country-specific variables. Some of the variables identified in this special issue include the differences in the health system (and payor system), the scope of roles that can be performed by genetic counselors, language, culture and religious differences, and even laws that may influence access to care (eg, abortion access). This also reminds us that solutions identified in one country may not be directly suitable for another.

We also reflect on how hard it is to ensure diversity within the workforce of genetic counseling providers. Most would agree it is ultimately desirable to have a workforce that reflects the population in regard to the important cultural, linguistic, and religious variables.^23^ However, even in countries with well-established genetic counselor professions, it remains a challenge to have a workforce reflecting the country’s diversity. In countries where genetic counseling is developing, there may not be training programs available within the country, or even in the language spoken within a given country. Genetic counselors trained elsewhere bring back practice models to one’s own or another country, facing the challenges described in matching cultural values and language during service delivery. In-country training programs, on the other hand, require support, funding, and locally available experts to teach and oversee clinical training. What can be achieved is also influenced by the resources available within the health system, such as translators and locally relevant information materials.

Finally, the articles in this special issue demonstrate that there is little consistency in data collected to document the professional spread of genetic counseling across the globe. Even the title of genetic counselor is used differently. This raises important questions: is there a minimal data set that professional organizations could collect to help standardize data about the profession? How could professional organizations better work together to collaborate and share resources, particularly so that countries with an emerging profession do not have to “reinvent the wheel” but rather can consider which approaches might be best suited to their local situations?

Perhaps now is the time to consider how genetic counseling professionals internationally might be mobilized to take this to the next level. Some genetic counselors over the years have been fortunate to work with international colleagues through the Transnational Alliance for Genetic Counselors, our regional professional societies, Human Genome Organization, or international meetings such as the World Congress of Genetic Counselling. These important but disparate fora have shown us the power of collaboration. Editing this special issue has even further convinced us of the need to join up the profession globally. A considered and coordinated approach is essential to address the challenges faced internationally, to ensure the integrity of our profession and its core values as it extends around the world, and to enhance how we learn from each other and evolve our practice locally. This special issue shows not only that there is a critical mass of activity but also, by virtue of its gaps and limitations, a pressing need.

An international professional organization (whether a society or federation) could be a reliable resource for professional standards endorsed by professional societies around the world, providing assistance to policy makers and educators in countries seeking to develop the profession of genetic counselors. For instance, in recent years, policy makers in countries wishing to establish genetic counseling have commonly experienced difficulty in deciding which training model and certification processes they should adopt. Universities have struggled to determine what curricular competencies to require when existing accreditation processes list long, aspirational lists of more than 75 content or skill areas. With a limited Master qualified genetic counselor workforce and urgent need to develop genetic counseling services, some countries prefer to recruit existing allied health professionals such as nurses or medical laboratory scientists as genetic counselors with short courses and/or on-the-job training provided. Trade-offs such as this can affect the genetic counseling profession in the long run. If countries lower the standards of training and quality required of genetic counselors permanently, rather than as a pragmatic first step, the result may be different standards around the world for how a genetic counselor is defined and the quality of practice. An international genetic counseling-focused organization would be well placed to address this concern.

In conclusion, we call on genetic counseling professional societies worldwide to work together to establish a structure that can and does fulfill a needed role. That is, to support those expanding the horizons of our profession, provide a platform for peer support, and ensure quality by serving as a reliable resource on professional standards. Through such an initiative, we can create a global organization to learn from each other, improve the understanding of genetic counseling practice between countries, and increase international research collaboration and cross-training within the international network.

## Conflict of Interest

The authors declare no conflicts of interest

## References

[1] Ormond K.E., Gaff C.L. (Published online July 17, 2023). Genetic counseling around the globe: a call for papers. Genet Med Open.

[2] Yashar B.M., Peterson M. (2013). Introduction to the special issue on genetic counseling: a global perspective. J Genet Couns.

[3] Abad P.J.B., Tumulak M.A.J.R., Yoon S.Y., Laurino M.Y., Hasan Q. (2024). Bibliometric analysis of genetic counseling publications in Asia: insights and implications. Genet Med Open.

[4] Weil J., Alaeddin D., Awwad R. (2024). Promoting international, locally focused, and patient-oriented genetic counseling. Genet Med Open.

[5] Ormond K.E., Abad P.J., MacLeod R., Nishigaki M., Wessels T.M. (2024). The global status of genetic counseling in 2023: what’s changed in the past 5 years?. Genet Med Open.

[6] Wessels T.M., Greenberg J.L., Fourie K., Kromberg J.G.R. (2024). Genetic counseling in South Africa: a growing profession. Genet Med Open.

[7] Kanga-Parabia A., Mitchell L., Smyth R. (2024). Genetic counseling workforce diversity, inclusion, and capacity in Australia and New Zealand. Genet Med Open.

[8] Schwaninger G., Heidemann S., Rudnik-Schöneborn S., Zschocke J. (2024). The current state of the genetic counselor profession in the German-speaking countries. Genet Med Open.

[9] Schwaninger G., Taxer K., Marti S. (2024). Workshop report: clinical training and integration of genetic counselors into interprofessional teams in the German-speaking countries. Genet Med Open.

[10] Abiib S., Khodjet-El-Khil H., El-Akouri K. (2024). Qatar’s genetic counseling landscape: current insights and future prospects. Genet Med Open.

[11] Al-Kharusi K., Van Wyk C., Al Hinai M., Al-Fori A., Bruwer Z. (2024). Genetic counseling development and milestone in Oman. Genet Med Open.

[12] Adzemovic T., Kabbale K.D., Katagirya E., Mukisa J., Wayengera M. (2024). “A Call To Action” – the need for genetic counseling in Uganda. Genet Med Open.

[13] Fernández-Ramires R., Morales-Pison S., Rucatti G.G. (2024). Cancer genetic counseling in Chile: addressing barriers, confronting challenges, and seizing opportunities in an underserved Latin American Community. Genet Med Open.

[14] Diaz Caro D., Simone L. (2024). The role of the Latin American professional society of genetic counseling (SPLAGen): advancing genetic counseling in Latin America. Genet Med Open.

[15] Furqan A., Ahmed S.A., Naeem R., Ashfaq M. (2024). Developing medical genetics in a low-income country: unveiling the journey of the Pakistani society of medical genetics and genomics (PSMG). G Genet Med Open.

[16] Altemura C., Diaz Caro D., Fife N., Giacomazzi V., Sumarroca M. (2024). The impact of English-centric training for multilingual genetic counseling practice: a commentary. Genet Med Open.

[17] Jadeja N., Rajakumar N., Reddy N., Ali N., Lichten L. (2024). Reflections on my international genetic counseling rotations: contrasts in practice between India and the United States. Genet Med Open.

[18] Valverde K.D., Hartman T.R., Reichert S.L. (2024). Continuing education and professional development: unifying opportunities for genetic counselors globally. Genet Med Open.

[19] Canton H., Macintosh R., Sweeting J. (2024). A mixed-methods assessment of the Australasian Society of Genetic Counselors (ASGC) Mentor Program. Genet Med Open.

[20] Feufel M.A., Speiser D., Schüürhuis S. (2024). iKNOW – supporting the counselling of women with hereditary risk of breast and ovarian cancer with digital technologies: a randomized controlled trial. Genet Med Open.

[21] Chávarri-Guerra Y., Rodríguez-Olivares J.L., Ramírez-González A. (2024). Addressing the need for genetic cancer risk assessment in Mexico: from establishment of a formal program to delivery innovation and expansion. Genet Med Open.

[22] Boggs K., Lynch F., Ward M. (2024). Rapid genomic testing in critically ill pediatric patients: genetic counseling lessons from a national program. Genet Med Open.

[23] Gomez L.E., Bernet P. (2019). Diversity improves performance and outcomes. J Natl Med Assoc.

